# Fast Sparse Coding for Range Data Denoising with Sparse Ridges Constraint

**DOI:** 10.3390/s18051449

**Published:** 2018-05-06

**Authors:** Zhi Gao, Mingjie Lao, Yongsheng Sang, Fei Wen, Bharath Ramesh, Ruifang Zhai

**Affiliations:** 1 Temasek Laboratories, National University of Singapore, 117411 Singapore; tsllaom@nus.edu.sg (M.L.); tslrame@nus.edu.sg (B.R.); 2 College of Computer Science, Sichuan University, Chengdu 610065, China; sangys@scu.edu.cn; 3 School of Remote Sensing and Information Engineering, Wuhan University, Wuhan 430079, China; wenfei@whu.edu.cn; 4 College of Informatics, Huazhong Agricultural University, Wuhan 430070, China; rfzhai@mail.hzau.edu.cn

**Keywords:** LiDAR, range data denoising, sparse coding, ridge constraint

## Abstract

Light detection and ranging (LiDAR) sensors have been widely deployed on intelligent systems such as unmanned ground vehicles (UGVs) and unmanned aerial vehicles (UAVs) to perform localization, obstacle detection, and navigation tasks. Thus, research into range data processing with competitive performance in terms of both accuracy and efficiency has attracted increasing attention. Sparse coding has revolutionized signal processing and led to state-of-the-art performance in a variety of applications. However, dictionary learning, which plays the central role in sparse coding techniques, is computationally demanding, resulting in its limited applicability in real-time systems. In this study, we propose sparse coding algorithms with a fixed pre-learned ridge dictionary to realize range data denoising via leveraging the regularity of laser range measurements in man-made environments. Experiments on both synthesized data and real data demonstrate that our method obtains accuracy comparable to that of sophisticated sparse coding methods, but with much higher computational efficiency.

## 1. Introduction

In addition to their increasing proliferation and the growing importance of their roles in numerous applications (including 3D reconstruction and landscape surveying), light detection and ranging (LiDAR) sensors have recently been widely deployed on intelligent systems such as unmanned ground vehicles (UGVs) and unmanned aerial vehicles (UAVs) to perform localization, obstacle detection, and navigation tasks. Consequently, research into range data processing with competitive performance in terms of both accuracy and efficiency has attracted increasing attention. Despite the simplicity and directness in obtaining 3D information, such range data often suffer from noise due to the reflectance property of an object’s surfaces, or due to electrical and mechanical disturbances. Therefore, efficient denoising for range data remains a critical problem.

Some range data denoising methods directly originate from their counterparts for images by regarding the depth value of range data as intensity, and readers can refer to [1] for detailed discussions. However, such methods typically cause artifacts of vertex drifting, which reduce the geometric regularity [2,3]. Techniques designed specifically for range data (e.g., the 3D points cloud) can be roughly classified into point-based [4,5] and mesh-based [6,7] methods. However, estimating normal vectors from noisy data for mesh denoising appears to be a chicken-and-egg problem and remains an open topic [8,9]. In [10,11], range data and color images were fused for denoising, and promising results were reported. However, the prerequisites of these methods only hold when expensive sensors are applied or extra efforts are devoted to registering range data and images.

Sparse coding (SC) has revolutionized signal processing and led to state-of-the-art performance in a variety of applications, including image or video denoising, inpainting [12,13], restoration [14], and synthesis [15], face recognition [16], and anomaly detection [17]. These successes are mainly due to the fact that signal (images or image patches) have naturally sparse representations with respect to appropriate bases [18]. In recent years, such SC techniques have been applied to range data [19,20] and obtained competitive results. However, dictionary learning, which plays the central role in SC techniques, is computationally demanding, resulting in its limited applicability in real-time systems [21,22].

Based on the work of [20], in which reflectance information was synergized with depth data to facilitate the enforcement of adaptive sparsity constraint (therein, the depth and reflectance information that are complementary to each other work together to estimate the informative level of each patch, followed by adaptive dictionary learning which assigns more atoms to represent the patches with more information), we propose SC algorithms without dictionary learning to realize range data denoising via leveraging the regularity of laser range measurements in man-made environments. Specifically, a second-order differential transformation is applied to extract ridges that can be reconstructed and enhanced using a pre-learned ridge dictionary with sparsity constraint. Instead of performing refinement on the coefficients and dictionary alternately and iteratively [12,14,19,20,22], our method can achieve a one-step closed-form solution, resulting in much improved efficiency. Experiments on both synthesized data and real data were conducted to verify the effectiveness of our method, together with performance comparison against other state-of-the-art methods.

## 2. Our Method

### 2.1. Notation and Preliminaries

Matrices and vectors are shown in bold capital and bold lower-cased fonts, respectively. Sets are denoted with calligraphic fonts (e.g., S), and their cardinality is denoted as |S|. For a vector $x\in R^{m}$, x[i] is the *i*-th entry, $x_{S}$ is the subvector of x corresponding to the entries with indices in S. We define $\ell_{2}$-norm, ${\left||x|\right|}_{2}=\sum_{i=1,\cdots ,m}x{\left[i\right]}^{2}$; $\ell_{1}$-norm, ${\left||x|\right|}_{1}=\sum_{i=1,\cdots ,m}\left|x\left[i\right]\right|$; $\ell_{0}$-norm, ${\left||x|\right|}_{0}\;=\;\left|supp\left(x\right)\right|$ (i.e., the number of nonzero elements). sign(x) is a sign vector with entries: sign(x[i])=1 if x[i]>0, sign(x[i])=−1 if x[i]<0, and zero otherwise. Based on the sign operator, we define the shrinkage operator:

$$\begin{array}{c} T_{\alpha }\left(x\right)\left[i\right]={\left(|x\left[i\right]|-\alpha \right)}_{+}sign\left(x\left[i\right]\right)=max\left(|x\left[i\right]|-\alpha ,0\right)sign\left(x\left[i\right]\right). \end{array}$$

For a matrix $X\in R^{m\times n}$ and an index set S, $X_{S}$ is the submatrix containing only the rows of X with indices in S. vec(X) denotes the column-wise vectorization of matrix X. Letters *d* and *r* in superscript refer to the components related to the depth and reflectance information, respectively. Letters *v* and *h* in subscript refer to the components related to the vertical and horizontal directions, respectively.

**Figure 1:**
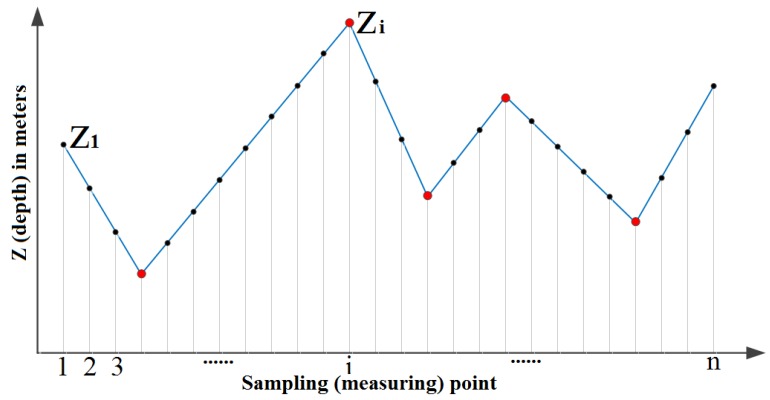
Illustration of the depth profile of a 2D laser scan.

**Ridge detection for range measurements**. We first take a scan from a 2D laser scanner as an example to introduce our operator of ridge detection and then extend the approach to 3D depth profiles. In Figure 1, we plot the depth profile $z\in R^{n}$ of a typical indoor environment, which is obtained using a standard planar range finder measured with (known) discrete angles. Clearly, the profile is sufficiently regular and contains a few corners. Considering three consecutive points at coordinates $\left(x_{i-1},z_{i-1}\right)$, $\left(x_{i},z_{i}\right)$, and $\left(x_{i+1},z_{i+1}\right)$, there is a corner at *i* if $\frac{z_{i+1}-z_{i}}{x_{i+1}-x_{i}}\neq \frac{z_{i}-z_{i-1}}{x_{i}-x_{i-1}}$. In the following, we assume that $x_{i}-x_{i-1}\;=\;1$ for all *i* (this assumption comes without loss of generality since we can define it at arbitrary resolution). Therefore, the corner detection for the 2D profile is to find those indices *i* such that $z_{i-1}-2z_{i}+z_{i+1}\neq 0$. To make the notation more compact, we introduce the second-order difference operator:

(1) $$D_{2nd}=\left[\begin{array}{cccccc} 1 & -2 & 1 & 0 & \cdots & 0\\ 0 & 1 & -2 & 1 & \cdots & 0\\ \vdots & 0 & \ddots & \ddots & \ddots & 0\\ 0 & \cdots & 0 & 1 & -2 & 1 \end{array}\right]\in R^{(n-2)\times n}.$$

Thus, a simple operation $D_{2nd}\cdot z$ can detect corners. For a 3D depth profile $Z\in R^{r\times c}$, we estimate the ridges of vertical and horizontal directions $R_{v}$ and $R_{h}$, respectively:

(2) $$\begin{array}{c} R_{v}=D_{v2nd}\cdot Z\in R^{(r-2)\times c},\\ R_{h}=Z\cdot D_{h2nd}^{T}\in R^{r\times (c-2)}, \end{array}$$

where the matrices $D_{v2nd}$ and $D_{h2nd}$ are the same as $D_{2nd}$ in Equation (1), but with suitable dimensions. To combine the two equations of (2), we reformulate it as below:

(3) $$\left[\begin{array}{c} vec(R_{h})\\ vec(R_{v}) \end{array}\right]=\left[\begin{array}{c} I_{c}⊗D_{v2nd}\\ D_{h2nd}⊗I_{r} \end{array}\right]\cdot vec\left(Z\right)=D_{vh}\cdot vec\left(Z\right).$$

Here, ⊗ is the Kronecker product, and $D_{vh}\in R^{2(r\cdot c-r-c)\times (r\cdot c)}$.

### 2.2. Adaptive SC and Its $\ell_{0},\ell_{1}$ Solutions

**Figure 2:**
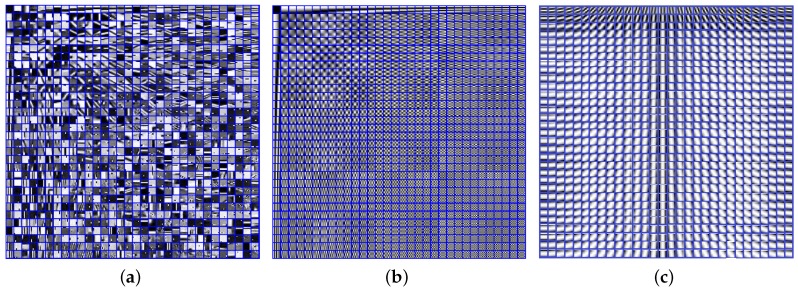
Our pre-learned dictionary of ridge maps (**a**), discrete cosine transform (DCT) dictionary (or say basis) (**b**), and Gabor wavelet dictionary (or say basis) (**c**).

For each patch $r_{i}$ (in the vectorized form) in the depth ridge map, we formulate the following adaptive SC model:

(4) $$\begin{array}{cc} \underset{x_{vi},x_{hi}}{min} & {\left|\left|r_{vi}-D_{L}\cdot x_{vi}\right|\right|}_{2}+{\left|\left|r_{hi}-D_{L}\cdot x_{hi}\right|\right|}_{2},\\ \;\;\;s.t.\; & \forall 1⩽i⩽N,\;{\left|\left|x_{vi}\right|\right|}_{0}⩽k_{vi},\;{\left|\left|x_{hi}\right|\right|}_{0}⩽k_{hi}, \end{array}$$

where $r_{vi}$ and $r_{hi}$ represent the patches from vertical and horizontal ridge maps, respectively. $D_{L}$ is the fixed pre-learned dictionary, as shown in Figure 2, which is obtained by applying the basic SC algorithm [19] on 40 pre-selected ridge maps of man-made scenarios (here, we display our pre-learned dictionary with the optimal setting of parameters as obtained in Section 3.1.1, namely the number of dictionary atoms is 1024 and the size of each atom is 8 × 8). In [23], different dictionaries (including off-the-shelf ones such as discrete cosine transform (DCT) basis, wavelets basis, and dictionaries obtained via learning—either pre-learned or learned from the data itself) were tested for image denoising. Although the dictionary learned from the data itself reported the best denoising accuracy, it was also the most computationally demanding. In addition to our pre-learned dictionary, we also displayed the DCT dictionary, Gabor wavelets dictionary in Figure 2. The parameter tweaking of such dictionaries and their denoising performance are discussed in Section 3.1.1. $x_{vi}$ and $x_{hi}$ correspond to the sparse coefficient vectors. $k_{vi}$ and $k_{hi}$ are the given sparsity controlling parameters. Instead of setting these parameters as constants for all patches, we set them adaptively according to the informative level of each patch (see Section 3.1.1 for details) in a manner similar to that of [20]. Given $k_{vi}$ and $k_{hi}$, the popular orthogonal-matching-pursuit (OMP) algorithm is applied to solve such $\ell_{0}$ optimization problem. Moreover, the batch-OMP (BOMP) technique is exploited to further improve the efficiency. As the problem of (4) is non-convex and NP-hard, we reformulate its relaxation as below:

(5) $$\begin{array}{cc} \underset{x_{vi},x_{hi}}{min} & {\left|\left|r_{vi}-D_{L}\cdot x_{vi}\right|\right|}_{2}+\lambda_{vi}{\left|\left|x_{vi}\right|\right|}_{1}\\ + & {\left|\left|r_{hi}-D_{L}\cdot x_{hi}\right|\right|}_{2}+\lambda_{hi}{\left|\left|x_{hi}\right|\right|}_{1}. \end{array}$$

Here, $\lambda_{vi}$ and $\lambda_{hi}$, which balance the representation fidelity term and the sparsity penalty term, are again estimated adaptively (see Section 3.1.1 for details). By stacking all the patches, we obtain Equation (6):

(6) $$\begin{array}{cc} \underset{X_{v},X_{h}}{min} & {\left|\left|\left[r_{v1},\cdots ,r_{vN}\right]-D_{L}\cdot X_{v}\right|\right|}_{2}+\sum_{i=1}^{N}\lambda_{vi}{\left|\left|x_{vi}\right|\right|}_{1}\\ + & {\left|\left|\left[r_{h1},\cdots ,r_{hN}\right]-D_{L}\cdot X_{h}\right|\right|}_{2}+\sum_{i=1}^{N}\lambda_{hi}{\left|\left|x_{hi}\right|\right|}_{1}, \end{array}$$

in which the neighboring patches are extracted with half-overlapping. To solve such $\ell_{1}$ optimization problem, the least-absolute-shrinkage-and-selection-operator (LASSO) algorithm was proposed, which has proven to find the global optimizer.

Clearly, Equations (4)–(6) differ from previous methods [12,14,19,20,22] in one aspect, which is that $D_{L}$ is fixed. Thus, $X_{v}$ and $X_{h}$ can be obtained with a one-step closed-form solution instead of performing refinement on the coefficients and dictionary alternately and iteratively, thus resulting in much improved efficiency. With $X_{v}$ and $X_{h}$ in hand, we reconstruct each patch ${\hat{r}}_{vi}$ and ${\hat{r}}_{hi}$. When all patches are processed, the average value is used for the locations occupied by multiple patches, and then we obtain the refined ridge maps ${\hat{R}}_{v}$ and ${\hat{R}}_{h}$. Next, we can recover $\hat{Z}$ by performing the inverse operation of Equation (3). However, due to the dependency of the rows of $D_{vh}$, the system is under-determined. Therefore, we need to incorporate the boundary conditions to recover $\hat{Z}$ from its refined second-order difference, as shown in Equation (7):

(7) $$\left[\begin{array}{c} z_{N}\\ vec(R_{h})\\ vec(R_{v}) \end{array}\right]=\left[\begin{array}{c} I_{N}\;0_{N\times (r\cdot c-N)}\\ D_{vh} \end{array}\right]\cdot vec\left(Z\right)=\left[\begin{array}{c} A\\ D_{vh} \end{array}\right]\cdot vec\left(Z\right),$$

where the available *N* boundary points are incorporated, and six boundary points are used in our work. A more rigorous theoretical proof of the minimum necessary boundary points is out of the scope of this work. Performing the inverse operation on (7), we can recover $\hat{Z}$ as Equation (8):

(8) $$vec\left(\hat{Z}\right)={\left[\left[\begin{array}{c} A^{T}\;D_{vh}^{T} \end{array}\right]\left[\begin{array}{c} A\\ D_{vh} \end{array}\right]\right]}^{-1}\left[\begin{array}{c} A^{T}\;D_{vh}^{T} \end{array}\right]\left[\begin{array}{c} z_{N}\\ vec(R_{h})\\ vec(R_{v}) \end{array}\right].$$

Here, all the matrices A, $D_{vh}$, their transposes, and their inverses can be pre-computed to significantly reduce the overall processing time. We now summarize all previous operations as Algorithm 1. In fact, our algorithm includes two versions of implementation: *our-BOMP* and *our-LASSO*.

In Algorithm 2, the convergence criterion is well-defined as ${\left|\left|x\right|\right|}_{0}⩽k$. In Algorithm 3, the convergence criterion is that the relative update difference of $x_{k}$ is less than $10^{-3}$.

**Algorithm 1** Our Sparse Coding for Depth Data Denoising
 **Input:**Depth map Z, dictionary $D_{c}$, parameters $k_{1}$, $k_{2}$, $\lambda_{v}$, $\lambda_{h}$; **Output:**Restored $\hat{Z}$ of Equation (8);1:Estimate the ridge maps $R_{V}$ and $R_{H}$ as Equation (2);2:Extract and vectorize ridge map patches $r_{vi}$, $r_{hi}$, 1⩽i⩽N;3://Line 5 & 7 apply different algorithms to estimate $x_{vi}$, $x_{hi}$.4://Line 5 belongs to the version of *our-BOMP*.5:Apply the Batch-OMP algorithm to solve Equation (4); 6://Line 7 belongs to the version of *our-LASSO*.7:Apply the LASSO algorithm to solve Equation (5); 8:Obtain the refined ridge maps ${\hat{R}}_{v}$ and ${\hat{R}}_{h}$ via reconstruction; 9:Recover $\hat{Z}$ via applying Equation (8).


**Algorithm 2** Batch-orthogonal-matching-pursuit [24] to solve: ${min}_{x}{\left|\left|y-D\cdot x\right|\right|}_{2}\;\;s.t.\;{\left|\left|x\right|\right|}_{0}⩽k$
 **Input:**y, D, *k*; **Output:**x;1:Initialization: x=0, r=y, S={⌀};2:**while** stopping criterion not met **do**3:$k={argmax}_{k}\left|d_{k}^{T}\cdot r\right|$;4:L=S;5:S={k}+L;6:$G_{S}=D_{S}^{T}D_{S}$, $G_{L}=D_{L}^{T}D_{L}$;7://Line 8 obtains $G_{S}^{-1}$ using progressive Cholesky update.8:$x_{S}=G_{S}^{-1}D_{S}^{T}y$;the key updating function to estimate $G_{S}^{-1}$ is: $$G_{S}^{-1}\;=\;\left[\begin{array}{cc} G_{L}^{-1}+\frac{1}{a}G_{L}^{-1}D_{L}^{T}d_{k}d_{k}^{T}D_{L}G_{L}^{-1} & -\frac{1}{a}G_{L}^{-1}D_{L}^{T}d_{k}\\ -\frac{1}{a}d_{k}^{T}D_{L}G_{L}^{-1} & \frac{1}{a} \end{array}\right]$$ here, $a=d_{k}^{T}d_{k}-d_{k}^{T}D_{L}G_{L}^{-1}D_{L}^{T}d_{k}$9:$r=y-D_{S}x_{S}$;10:
**end while**



**Algorithm 3** Fast iterative shrinkage-thresholding algorithm (FISTA) [25] to solve LASSO problem: $\min_{x}{\left|\left|y-D\cdot x\right|\right|}_{2}+\lambda {\left|\left|x\right|\right|}_{1}$
 **Input:**y, D, λ; **Output:**x;1:Initialization: $x_{0}=0$, $z_{1}=x_{0}$, $L=max(eig\left(D^{T}D\right))$, $t_{0}=1$, k=0;2:**while** not converged **do**3:k=k+1;4:$grad\left(z_{k}\right)=D^{T}Dz_{k}-D^{T}y$;5:$x_{k}=T_{\frac{\lambda }{L}}\left(z_{k}-\frac{1}{L}grad\left(z_{k}\right)\right)$;6://see Section 2.1 for the definition of $T_{\alpha }\left(x\right)$.7:$t_{k}=\left(1+\sqrt{1+4\cdot t_{k-1}^{2}}\right)/2$;8:$z_{k+1}=x_{k}+\frac{t_{k-1}-1}{t_{k}}\left(x_{k}-x_{k-1}\right)$;9:
**end while**



## 3. Experiments and Analysis

We performed experiments on both synthesized data and actual data recorded using a laser scanner. In the synthesized data experiments where the ground truth was available, the setting of important parameters was investigated, followed by denoising evaluation. In the real data experiments, the results are also demonstrated for visual assessment, in addition to the performance comparison. Similar to [14,20], the peak signal-to-noise ratio (PSNR) value is employed to evaluate the performance. Here, we clarify that our work is run on an ASUS Pro Notebook with a 2.4 GHz Intel Quad-Core i7-4500U processor and 12 GB RAM executing MATLAB code in parallel.

### 3.1. Experiments on Synthesized Data

**Figure 3:**
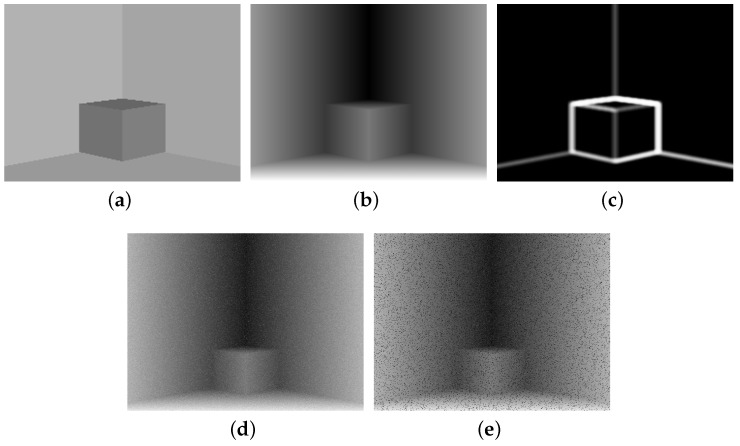
Synthesized data (of size 800×800 pixels) designed for experiments. (**a**) Reflectance image. (**b**) Depth map. (**c**) Result of informative-level estimation. (**d**) Gaussian noise-corrupted depth data. (**e**) Gaussian noise and outliers-corrupted depth data.

Similar to [20,26], we designed a scene of a square box having sides of one meter in a room, as shown in Figure 3, where all information is known as the ground truth.

#### 3.1.1. Important Parameters

To achieve plausible performance in terms of both accuracy and efficiency, the following parameters play the central role: the sparsity controlling parameters $k_{vi}$, $k_{hi}$, $\lambda_{vi}$, $\lambda_{hi}$, dictionary dimension *d* (which indicates that the patch size is $\sqrt{d}\times \sqrt{d}$ pixels), and its atom number $n_{D}$ ($r=\frac{n_{D}}{d}$ is the redundancy of the dictionary).

Applying the same parameter tweaking strategy as that in [20], we recommend the sparsity controlling parameters as shown in Table 1. For each patch $P_{j}$ (j=v or *h*, from vertical or horizontal ridge maps, respectively), the parameters $\alpha_{vi},\beta_{vi}$ (or $\alpha_{hi},\beta_{hi}$) are related to its informative-level measurement (InM), which is estimated as below:

(9) $$\begin{array}{c} D_{en}\left(P_{v}^{d}\right)=\sum_{i=1}^{\sqrt{d}}\sum_{j=1}^{\sqrt{d}}\left|P_{v}^{d}\left[i,j\right]\right|,\\ R_{en}\left(P^{r}\right)=\sum_{i=1}^{\sqrt{d}}\sum_{j=1}^{\sqrt{d}}GradMag\left(P^{r}\right)\left[i,j\right],\;\;GradMag\left(P^{r}\right)\left[i,j\right]=\sqrt{{\left(\frac{\partial P^{r}\left[i,j\right]}{\partial x}\right)}^{2}+{\left(\frac{\partial P^{r}\left[i,j\right]}{\partial y}\right)}^{2}},\\ InM\left(P_{v}^{d}\right)=D_{en}\left(P_{v}^{d}\right)\cdot \exp\left(\sigma \left(R_{en}\left(P^{r}\right)\right)-0.5\right). \end{array}$$

Here, $GradMag(P^{r})$ is the gradient magnitude map of patch $P^{r}$, and σ(·) is the sigmoid function $\sigma \left(\cdot \right)=\frac{1}{1+exp(\cdot )}$. For the input from (−∞,+∞), the output of σ(·) is in the range (0,1). In our work, as the value of $R_{en}$ cannot be negative, the output of $\sigma (R_{en})$ is in the range [0.5,1). The output of $\exp(\sigma \left(R_{en}\left(P^{r}\right)\right)-0.5))$ is in the range $\left[1,e^{0.5}\right)$. With $InM\left(P_{v}^{d}\right),InM\left(P_{h}^{d}\right)$ for all patches in hand, we determine the sparsity controlling parameters via computing $\alpha_{vi},\beta_{vi},\alpha_{hi},and\beta_{hi}$ as below:

(10) $$\begin{array}{c} InM_{min}=min\left\{InM\left(P_{vi}^{d}\right),InM\left(P_{hi}^{d}\right)\right\},\;\;i=1,\cdots ,N,\\ InM_{max}=max\left\{InM\left(P_{vi}^{d}\right),InM\left(P_{hi}^{d}\right)\right\},\;\;i=1,\cdots ,N,\\ \alpha_{ji}=\frac{InM_{max}-InM\left(P_{ji}^{d}\right)}{InM_{max}-InM_{min}},\;\;\;j=v\;or\;h,\\ \beta_{ji}=\frac{InM\left(P_{ji}^{d}\right)-InM_{min}}{InM_{max}-InM_{min}},\;\;\;j=v\;or\;h. \end{array}$$

In Table 1, the positions of $\alpha_{ji}$ and $\beta_{ji}$ are exchanged in *Our-BOMP* and *Our-LASSO* because $k_{ji}$ and $\lambda_{ji}$ enforce the sparsity constraint in different manners to adaptively determine the number of dictionary atoms permitted for the patches with different informative levels. For example, to assign more atoms to represent the patch with more information, $k_{ji}$ should increase to push up the sparsity limit, whereas $\lambda_{ji}$ should decrease to penalize the sparsity constraint term to a lower degree.

**Figure 4:**
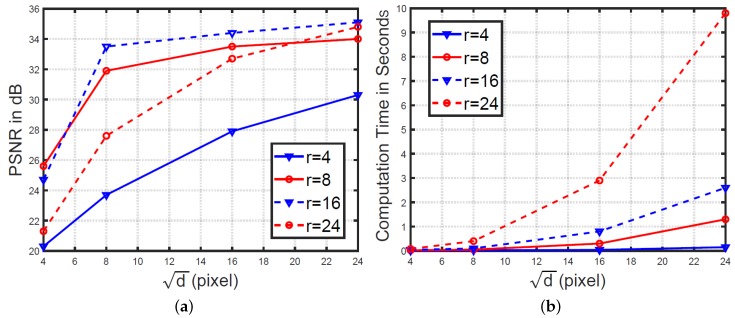
(**a**) PSNR and (**b**) computation time with dictionaries of different combinations of patch size and redundancy.

In addition to $k_{ji},\lambda_{ji}$, we tested different combinations of patch size and redundancy to find the most reasonable settings of *d* and *r*. Specifically, $\sqrt{d}$ can be 4, 8, 16, or 24, and *r* can be 4, 8, 16, or 24. Thus, a total of 16 combinations were tested. As shown in Figure 4a, dictionaries with redundancy 16 consistently achieved better denoising results than dictionaries with redundancy 4, 8, and 24, when their patch sizes were the same. Figure 4b shows the computation time. Clearly, the time increased dramatically with increasing patch size and redundancy. Taking both the denoising performance and computation time into account, we conclude that the dictionary setting of $\sqrt{d}$ = 8 and *r* = 16 (namely 1024 atoms) can yield decent outputs, while allowing fast computation. Such parameters are used throughout this paper, except where indicated. In Table 2, we further report the denoising result using different fixed dictionaries: our pre-learned dictionary, DCT dictionary, and Gabor wavelets dictionary. Clearly, the results using our pre-learned dictionary were better those using off-the-shelf ones.

#### 3.1.2. Denoising Results

The denoising results of our work on the synthesized data (see Figure 3d in which 100% dense Gaussian noises with standard deviation of 0.02 m and gray-scale value 5 for range and reflectance data respectively were added) are reported in Table 3, together with the comparison against available competitive methods including *Trilateral filter* [26], *Basic SC* [19], and *Adaptive SC* [20]. Moreover, in addition to the Gaussian noise, sparse outliers (5% of the total positions, which were randomly distributed) were added to the scene, as shown in Figure 3e, and the denoising results are also reported in Table 3. In addition to the PSNR criteria, the root mean square (RMS, with units of millimeters) error indicating the difference between the recovered range data and the ground truth is also included, since it can give readers a more direct indication of how well the contaminated range information was restored by these methods. Moreover, the computational time of each method is also reported. As shown in Table 3, our methods outperformed *Trilateral filter*, *Basic SC*, and obtained comparable results to *Adaptive SC* in terms of denoising accuracy (the best results were obtained by *Adaptive SC* with PSNR = 33.83 and RMS = 1.98), while achieving significantly higher efficiency than *Adaptive SC*. Moreover, *Our-BOMP* even worked slightly more efficiently than *Our-LASSO*, achieving the processing speed of approximately 13 fps for the range image with resolution 800 × 800.

### 3.2. Denoising Data from Laser Scanner

**Figure 5:**
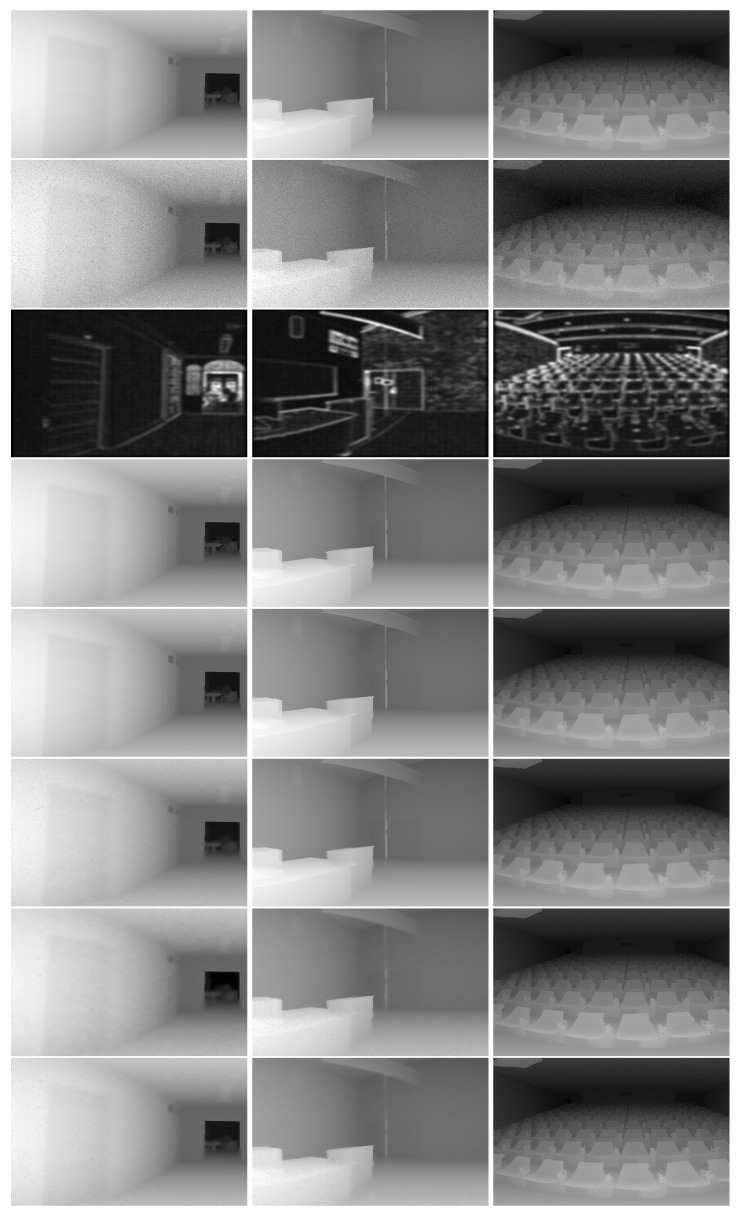
Results of denoising experiments on real data (see online version for details). The original and noisy corrupted range images are shown in the first and second rows, respectively. The third row depicts our intermediate results of informative level estimation. Rows 4 to 8 depict the results of ***Our-BOMP***, ***Our-LASSO***, ***Adaptive SC***, ***Basic SC***, and ***Trilateral filter***, respectively.

This set of experiments used the Brown range image database [27] captured with a Riegl LMS-Z210 laser scanner, its field of view (FoV) is $80^{\circ }$ vertically and $259^{\circ }$ horizontally. We focused only on the 15 indoor scenes (e.g., classrooms and theatres). To deal with (and benefit from) such a large horizontal FoV, similar to the multiple local projection method in [19], we partitioned the scene into several parts with $80^{\circ }$ FoV vertically and horizontally. Note that in the Brown range image database, the depth of each measuring point is saved sequentially, left to right and up to down. Thus, the image location only indicates the order of saving and it was necessary to perform local projection to obtain reasonable images. The consecutive parts were half-overlapping along the horizontal direction, then each part was projected to an image plane orientated to the center. Finally, the average of all the restored parts was computed to restore the whole scene. In the following, the denoising results on such real data are reported and discussed. Similar to Section 3.1, Gaussian noise was added to the depth and reflectance data, and the results on three representative scenes are shown in Figure 5.

As can be observed in Figure 5, the results of *Trilateral filter* were good in the planar regions, but contained a great deal of noise in the edge regions. Our method, *Basic SC*, and *Adaptive SC* are all based on SC, and obtained consistent results in all regions. Moreover, the results of our method in the third column were slightly worse than those of columns 1 and 2, because the curved structure of the scene in the third column posed a greater challenge to our sparse ridge assumption. In Table 4, the average denoising results on all 15 indoor scenes of the dataset are reported. Similar to the results on the synthesized data, our method outperformed *Trilateral filter* and *Basic SC* consistently, and obtained comparable results to those of *Adaptive SC* in terms of denoising accuracy, while achieving significantly higher efficiency than *Adaptive SC*. Compared with the results in Table 3, the results of Table 4 deteriorated as the structure of real scenes was more complex than the synthetic scene. Similar to the previous section, in addition to the Gaussian noise, sparse outliers were added to the scene, and the denoising results are also reported in Table 4.

## 4. Conclusions

In this study, based on our previous work [20], we propose SC-based algorithms in which a pre-learned ridge dictionary is applied to realize range data denoising by leveraging the regularity of laser range measurements in man-made environments. Experiments on both synthesized data and real data demonstrate that our method obtained accuracies comparable to those of sophisticated SC methods with much higher efficiency, achieving approximately 13 fps (for resolution of 800 × 800) with the resource of a lightweight laptop computer. Therefore, our method can be applied for real-time systems in man-made environments. Furthermore, we are trying to implement our method with GPU acceleration, intending to further increase the efficiency and also save the precious payload for unmanned systems, especially UAVs.

## Figures and Tables

**Table 1:** Setting of sparsity controlling parameters. BOMP: batch orthogonal-matching-pursuit; LASSO: least-absolute-shrinkage-and-selection-operator.

Parameters	Our–BOMP	Our–LASSO
	$k_{\mathrm{vi}},k_{\mathrm{hi}}$	$\lambda_{\mathrm{vi}},\lambda_{\mathrm{hi}}$
Max value	$k_{max}=6$	$\lambda_{max}=2.2$
Min value	$k_{min}=1$	$\lambda_{min}=0.2$
Adaptive value *	$k_{i}^{v}=\alpha_{i}^{v}k_{min}+\beta_{i}^{v}k_{max}$	$\lambda_{i}^{v}=\beta_{i}^{v}\lambda_{min}+\alpha_{i}^{v}\lambda_{max}$
	$k_{i}^{h}=\alpha_{i}^{h}k_{min}+\beta_{i}^{h}k_{max}$	$\lambda_{i}^{h}=\beta_{i}^{h}\lambda_{min}+\alpha_{i}^{h}\lambda_{max}$

* $\alpha_{i}^{v},\beta_{i}^{v},\alpha_{i}^{h},\beta_{i}^{h}$ are related to the informative estimation of each patch.

**Table 2:** Denoising results (peak signal-to-noise ratio (PSNR) value) using different dictionaries (unit: dB).

Dictionary	Dictionary with Different Dimensions		
	$r=10,\sqrt{d}=8$	$r=16,\sqrt{d}=8$	$r=16,\sqrt{d}=16$
DCT dictionary	32.26	32.37	32.17
Gabor Wavelet dictionary	32.46	32.58	32.92
Our pre-learned dictionary	32.86	**33.75**	**33.81**

**Table 3:** Denoising results on the synthesized data. RMS: root mean square; SC: sparse coding.

Method	Only Gaussian Noise			Gaussian Noise and Outliers		
	PSNR (dB)	RMS (mm)	Time * (s)	PSNR (dB)	RMS (mm)	Time * (s)
Our–BOMP	33.72	2.06	0.08	33.28	2.14	0.09
Our–LASSO	33.78	2.04	0.12	33.35	2.12	0.15
Adaptive SC	33.83	1.98	227.38	33.71	2.04	239.62
Basic SC	33.17	2.17	153.62	33.02	2.56	172.38
Trilateral filter	32.37	2.49	0.04	32.12	2.67	0.06

* **Time** indicates the computation time for each method.

**Table 4:** Denoising results on real data.

Method	Only Gaussian Noise			Gaussian Noise and Outliers		
	PSNR (dB)	RMS (mm)	Time * (s)	PSNR (dB)	RMS (mm)	Time * (s)
Our–BOMP	35.37	2.31	0.86	35.17	2.43	0.89
Our–LASSO	35.43	2.27	1.15	35.21	2.37	1.28
Adaptive SC	35.72	2.03	918.71	35.68	2.08	972.33
Basic SC	34.28	2.83	538.37	34.16	2.97	572.81
Trilateral filter	32.13	3.27	0.16	32.02	3.75	0.18

* **Time** indicates the computation time for each method.
